# Long-term follow-up of a large multilocular odontogenic keratocyst. Analysis of recurrences and the applied treatments

**DOI:** 10.4317/jced.62032

**Published:** 2024-09-01

**Authors:** Jordi Borrás-Ferreres, Iker Albisu-Altolaguirre, Cosme Gay-Escoda, Adalberto Mosqueda-Taylor

**Affiliations:** 1 DDS, MSc. Private practice in Oral Surgery and Implantology, Benicarló, Spain; 2 MD, DDS. Private practice in Oral Surgery and Implantology, San Sebastián, Spain; 3 MD, DDS, MSc, PhD, EBOS, OMFS. Chairman and Professor of the Oral and Maxillofacial Surgery Department, Faculty of Medicine and Health Sciences, School of Dentistry, University of Barcelona. Director of the Master’s Degree program in Oral Surgery and Implantology, EFHRE International University/FUCSO, Barcelona. Coordinator/Researcher of the IDIBELL Institute. Head of the Oral and Maxillofacial Surgery and Implantology Department of the Teknon Medical Center, Barcelona, Spain; 4 DDS, MSc. Professor of Oral Pathology and Medicine, Health Care Department, Metropolitan Autonomous University Xochimilco, Mexico City, Mexico

## Abstract

Recurrence is a well-known feature of odontogenic keratocyst (OKC). Compared with other odontogenic cysts, OKC is characterized by an infiltrating growth, aggressive biological behavior, and a greater tendency towards recurrence once removed, particularly when simple enucleation has been carried out. The recurrence rate is largely dependent upon the type of treatment applied; consequently, the planning of management must take into account the possible reasons why the cyst may recur. The present article describes the 25-year follow-up of a large multilocular OKC and discuss the possible causes of its multiple recurrences, as well as the treatments applied, with special attention on the last management approach adopted.

** Key words:**Odontogenic keratocyst, treatment, recurrences, long-term follow-up.

## Introduction

Odontogenic keratocysts (OKCs) are cystic jaw lesions that are classified as developmental cysts derived mainly from the dental lamina (1). They account for approximately 10-15% of all odontogenic cysts, with a mean patient age at onset of 35 years and a peak incidence in the third decade of life. These lesions are slightly more common in males than in females (proportion 1.3:1), and the majority (75%) are located in the lower jaw, with a predilection for the mandibular angle and ascending ramus (approximately 50%) (2).

Radiologically, most OKCs (85%) appear as a unilocular radiolucent lesion with well-defined margins and preservation of the cortical layer. Nevertheless, they may adopt a range of appearances, and a differential diagnosis with other lesions is therefore mandatory (3). Many unilocular lesions, particularly those of large size, show scalloped or lobulated margins, forming bone crests at the periphery (multilobular appearance); on the other hand, a few lesions (about 10%) are multilocular, with the cyst compartments being separated by genuine bony septae (4,5).

The growth pattern is characteristic, with marked mesiodistal spread and only very slight buccolingual expansion. In 70% of the cases tooth displacement occurs, and in 50% of the lesions cortical fenestration is observed, though their ability to produce root resorption is minimal (3).

Compared to other odontogenic cysts, OKCs exhibit an infiltrating growth pattern, an aggressive biological behavior and a greater tendency towards relapse following removal through enucleation (around 20-30%) (6,7). One of the possible reasons for this high rate of recurrence could be an incomplete removal of the cystic membrane, since its enucleation and curettage are known to be difficult, due to its thinness and friability (8). Another reason could be the persistence of satellite cysts or the presence of epithelial islands capable of forming new lesions within the cystic fibrous wall or located in the overlying oral mucosa (8,9).

Different surgical treatments for OKCs have been described (1,10-12). The most common strategy is enucleation/curettage, in which the lesion is removed in one or more fragments (6). This is followed in terms of frequency by marsupialization/decompression, which attempts to “decompress” the cyst by exposing its lumen and thus releasing the intracyst pressure. Marsupialization is a one-step procedure while decompression requires opening and placing an appliance (tube, etc.) to keep the cavity open until its size has decreased due to new bone forming at the periphery of the lesion. In either case, once the cyst has been decompressed, treatment is generally completed with enucleation of the lesion (13). Lastly, the most aggressive treatment option is resection, in which the entire lesion is removed along with a margin of adjacent unaffected bone that may or may not preserve continuity of the mandible (14). In addition to these primary treatments, there are other complementary methods that usually accompany enucleation/curettage to ensure complete elimination of the cyst and its possible satellite cysts or epithelial islands. The most widely used procedures are the Carnoy’s solution, 5-fluorouracil (5-FU), cryotherapy, peripheral ostectomy and excision of the overlying oral mucosa (9,11,15-17).

The present study describes the 25-year follow-up of a large multilocular OKC, the possible reasons for its recurrences and the treatments applied, with a detailed description of the last management approach adopted, based on different therapeutic variants trying to minimize the risk of future relapse.

## Case Report

**Figure 1:**
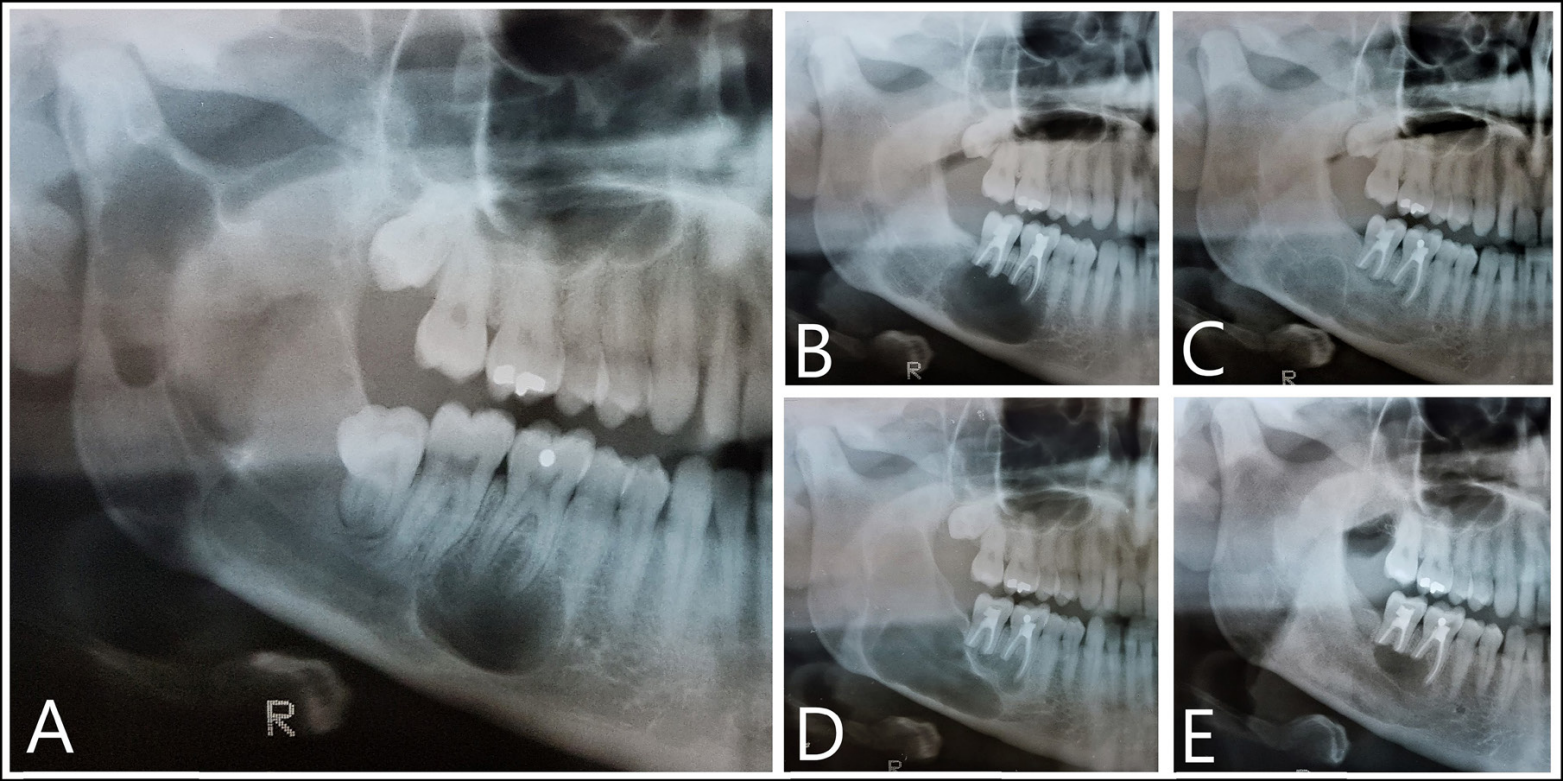
Evolution of the OKC during the first 12 years. A: April 2000 (diagnosis). B: November 2001. C: October 2003. D: January 2010. E: May 2012.

A 42-year-old male was incidentally diagnosed with a large multilocular OKC in April 2000, at the age of 20 years, on occasion of a control panoramic radiographic study. The lesion affected the entire right ascending ramus of the mandible, with involvement of the condyle and coronoid process, angle and body, extending to the zone of the mental foramen (Fig. 1A). The cyst did not displace the teeth or the inferior dental canal (IDC), and the affected molars remained vital. Treatment consisted of devitalization and apicoectomy of molars 4.6 and 4.7, the extraction of impacted tooth 4.8, and curettage of the cystic lesion. As a postoperative complication, the patient experienced paresthesia of the right inferior dental nerve that resolved completely in 6 months.

One year later a first recurrence was detected in the periapical zone of 4.6 and 4.7, and was allowed to evolve until buccal sulcus deformation was observed. At this point (November 2001), incision and drainage were carried out (Fig. 1B). Posterior follow-up showed the lesion to decrease in size. Two years later a second recurrence was observed at the angle of the mandible, together with suspected relapse in the zone of the first relapse (third recurrence) (October 2003) (Fig. 1C). The patient rejected further surgery and so the lesions were allowed to evolve freely for 7 years, when the size of the second recurrence became notorious (January 2010) (Fig. 1D). Treatment consisted of repeat curettage with amplification of the apicoectomies of the molars. The subsequent controls showed healing of the second recurrence, though with recurrence in the zone of the third relapse (fourth recurrence) (May 2012) (Fig. 1E). The patient again rejected surgery, and periodic observation was decided, showing the relapse to be in a latent phase.

**Figure 2:**
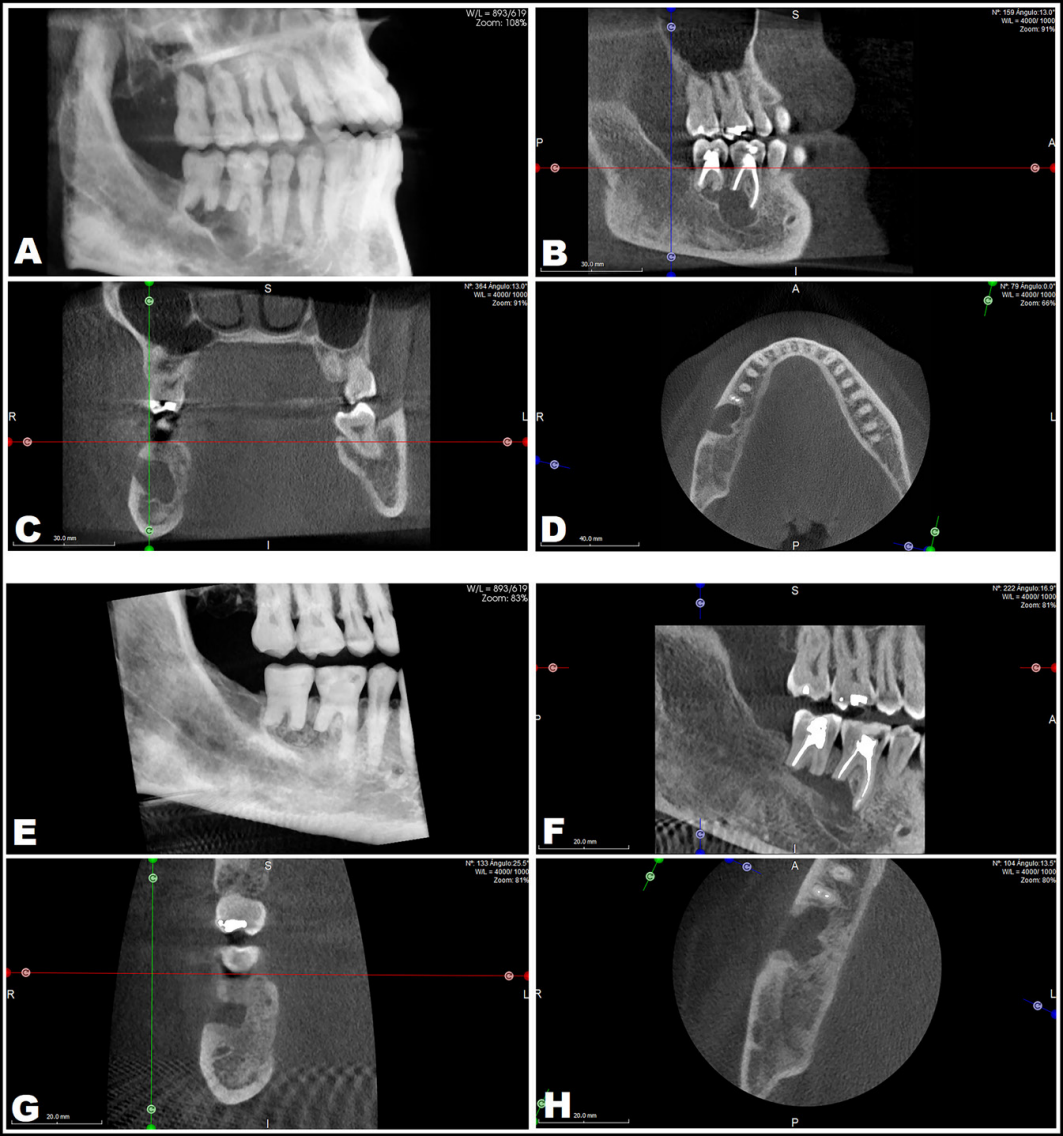
Cone-beam computed tomography scan. Situation before marsupialization. December 2019 (A-D). Situation 9 months after marsupialization. September 2020 (E-H).

Seven years later the patient again accepted treatment (December 2019). Cone-beam computed tomography (CBCT) revealed a multilobular lesion with radiopaque margins, in contact with the IDC and showing fenestration of the buccal cortical layer (Fig. 2A-D). Treatment was applied in two phases. Firstly, marsupialization with exeresis of the oral mucosa overlying the bony fenestration was carried out in order to reduce the size of the lesion and move it away from the IDC. Nine months later (September 2020), after CBCT confirmation of successful decompression (Fig. 2E-H), curettage was performed combined with a peripheral ostectomy and the application of Carnoy’s solution during 5 minutes. The decision was also made to remove the molars, since 4.6 presented asymptomatic root fracture, and 4.7 did not have a good long-term periodontal prognosis.

**Figure 3:**
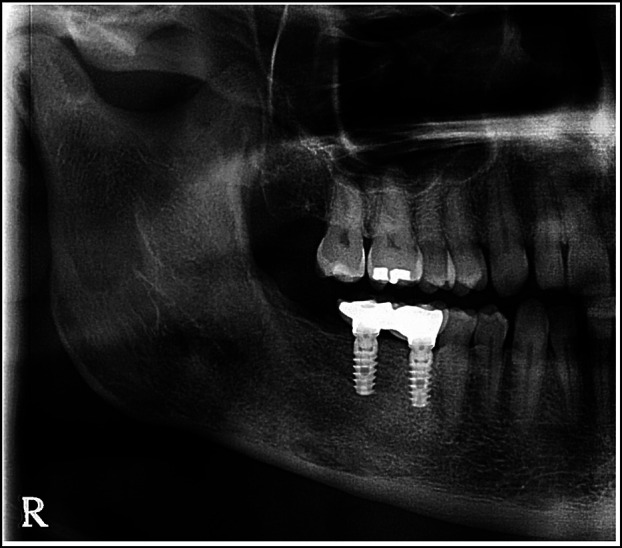
Evolution of the OKC. June 2024.

Over the next three years, the annual radiological controls showed good healing of the defect, with no signs of recurrence. The two molars were thus finally replaced with osteointegrated endosseous implants. The radiographic control at the present time, almost four years after last treatment of the OKC, continues to shown no evidence of disease (Fig. 3).

## Discussion

In the same way as some odontogenic tumors, OKCs arise from different potential epithelial sources, such as the prefunctional dental lamina (epithelium able to form teeth), which tends to be more abundant distal to the third molar in patients who have completed odontogenesis, as well as the post-functional dental lamina, which includes the so-called epithelial rests of Serres within the gingival fibrous tissue, and other free rests located at intra-osseous level; in addition, these cysts may also arise from basal cells of gingival epithelium, from which the dental lamina is originally derived (18).

The main mechanism of growth of these lesions involves epithelial proliferation, demonstrated by the increased mitotic activity of their lining, which explains the growth pattern and its effects upon the adjacent structures. In fact, a comparative study carried out by Brito-Mendoza *et al*. (19) showed the expression of Ki-67 (a proliferation marker) to be significantly higher in OKCs as compared to unicystic ameloblastomas. On the other hand, these authors also showed that the expression of syndecan-1 (CD138) (used as a cell adhesion marker) was also elevated, thus reflecting the scant invasive capacity of these lesions compared with unicystic ameloblastomas. Since OKCs lack a significative internal pressure (which is the main expansion factor of dentigerous and radicular cysts), the continuous growth of their walls secondary to the epithelial proliferation gives rise to evaginations that penetrate into the adjacent medullary space, excavating the route of least resistance to infiltration (20); therefore, the greater or lesser density of the alveolar cortical bone and IDC explains the absence or presence of displacement, respectively, of the teeth and trajectory of the inferior dental nerve. This growth pattern, together with scant osteoclastic activity, explains the multilobular/multilocular radiographic appearance of some cases (4,5).

According to Stoelinga (4), OKCs can present radiographically as unilocular, scalloped, multilobular or multilocular lesions. The multilocular radiographic pattern observed in our patient, together with the absence of tooth displacement of the affected teeth and of the IDC, suggests a relatively high density of the mandibular cancellous bone and of the alveolar cortical bone. This is quite probable, considering that the patient presented bruxism. On the other hand, mention has to be done of the radiotransparency located in the periapical zone of 4.6 at the time of diagnosis which, due to its intensity, probably corresponded to fenestration of the buccal/lingual cortical layers.

Although approximately 25% of all sporadic OKCs and 50% of those associated to Gorlin-Goltz syndrome present small “satellite” cysts in their fibrous wall (21), histologically manifesting as multicystic/multilocular lesions, it should be noted that this phenomenon cannot be seen on a radiograph. We therefore consider that the radiographic description of “multilocular” is not fully appropriate, and could give the impression that the lesion is composed of true multiple independent bony cavities. In this regard, the difficulty of radiographic interpretation is due to the uneven growth of the cyst, with a tendency to infiltrate areas of lesser tissue resistance (medullary spaces of cancellous bone). Consequently, from a radiographic point of view, the septae that seem to separate the cyst into various cavities are actually bony wall partitions produced by non-uniform bone reabsorption, and thus the cyst “cavities” in fact correspond, in the majority of cases, to a single space or cavity with multiple compartments. Thus, the radiographic “cavities” could also be due to cortical fenestration phenomena. The possibility of encountering totally independent cavities due to multiple independent OKCs located very close together seems to be much greater in patients with Gorlin-Goltz syndrome.

Although OKCs constitute a benign condition, a number of their biological characteristics define them as aggressive lesions, being able even to recur in autologous bone grafts placed after maxillary bone resection (12,14,22). It is important to identify these cysts, since they can reach a very large size without causing symptoms, as in our patient. Another aspect of interest is that around 5% of these lesions are associated to Gorlin-Goltz syndrome (nevoid basal cell carcinoma syndrome) and tend to relapse more frequently after treatment (3,5,13,23). In order to understand the possible causes of OKC relapse, we first must know the underlying etiopathogenesis. In addition to the dental lamina as a source of these cysts, another theory suggests that particularly in patients with Gorlin-Goltz syndrome, the lesions originate from projections derived from the epithelium of the oral mucosa towards the connective tissue (also known as basal cell hamartias) (24). These two potential sources of OKCs are not incompatible, since both the dental lamina and basal cell hamartias share the same ectodermal origin. With regard to recurrences, Voorsmit *et al*. (8) point out the following three possible causes:

• The persistence of some cyst fragments. This is a real possibility, due to the thinness and great friability of the cystic membrane. The high mitotic activity of the basal and suprabasal cells of the residual epithelium would give rise to new cysts developing from the fragments remaining in the surgical bed (19,20). On the other hand, since the junction of the epithelium with the connective tissue capsule is very weak because of the destructive action of collagenases produced there, the epithelium easily becomes detached during surgery and may be reimplanted in the surgical bed or in neighboring tissues, giving rise to a recurrence (1).

• The growth of a satellite cyst located in the wall of the lesion (21). It is still not clear whether the former is produced by active proliferation of the basal layer of the cyst epithelium or develops from the original epithelial source that also produced the main cyst. Despite complete elimination of the original epithelial lining, the persistence of a satellite cyst located within the connective tissue wall, whether in the bone cavity or in the surrounding tissues, would explain future recurrences.

• “Recurrence” in fact would be a new OKC developing in the vicinity of the old lesion. Like the original cyst, this new cyst would have developed from remains of the dental lamina or from epithelial projections (hamartias) produced by basal cells of the overlying oral mucosa – a situation more often seen in patients with Gorlin-Goltz syndrome (24).

In relation to treatment, curettage is the most commonly used strategy, since complete enucleation is very complicated because of the shape and friability of the cyst tissue (16). This explains why enucleation as sole treatment strategy is associated with very high rates of recurrence. In their meta-analysis, Slusarenko da Silva *et al*. (7) reviewed 6 studies reporting a total of 202 OKCs. The recurrence rate of the lesions that had been enucleated was 28.9% (n = 135) versus 16.4% (n = 67) when enucleation was preceded by up to 23.5 months of marsupialization or decompression. On the other hand, resection is the management strategy with the lowest relapse rates, though it can produce considerable mutilation (14,25). In addition to these management strategies, efforts have been made to define an intermediate complementary treatment affording an acceptable long-term success rate with less morbidity. The complementary therapeutic options include decompression/marsupialization, the use of Carnoy’s solution, the application of 5-FU, cryotherapy, peripheral ostectomy, and excision of the overlying oral mucosa (9,11,15-17).

• Decompression/marsupialization is performed to create a window in the wall of the cyst, exposing its contents and maintaining continuity of its surface with the oral cavity. The only portion of the cyst membrane that is removed corresponds to the tissue that is separated for the creation of the window, while the rest is left in place (13). This technique reduces the size of the cyst and induces epithelial metaplasia that modifies the histology, thickens the fibrous wall and improves the prognosis (1,10,26). The application of this procedure greatly facilitates enucleation and detachment from neighboring anatomical structures, reducing the risk of iatrogenic effects in subsequent resection. The systematic review carried out by Tabrizi *et al*. (23) included 192 OKCs subjected only to marsupialization (n = 118) or to marsupialization or decompression followed a few months later by enucleation (n = 64), and they recorded recurrence rates of 27.1% and 10.9%, respectively. The difference between these approaches was that in decompression a smaller window was made and kept open with a surgical drain sutured to the margins, with gradual reduction as the size of the lesion decreased. In contrast, marsupialization involved a larger window that was kept open with surgical dressing (13).

• Due to the frequent persistence of cyst fragments in the surrounding bone, peripheral ostectomy is recommended (1-2 mm); to increase the area that can be free of epithelial remnants chemical fixation may be performed by applying Carnoy’s solution or 5-FU. Liquid nitrogen cryotherapy also has been used. These treatments denaturalize the cells located at a depth of 1-2 mm in the bone surrounding the cystic lesion (11,15,17). Zhao *et al*. (25) reported a recurrence rate of 17.8% in patients subjected to enucleation versus 6.7% in those subjected to enucleation followed by the application of Carnoy’s solution.

• Excision of the oral mucosa overlying the bone fenestration in continuity with the cyst can be performed with the aim of eliminating possible epithelial islands or satellite cysts located in that area (9,24).

Regarding the present case, it should be mentioned that the treatments employed and follow-up from diagnosis in the year 2000 until December 2019 were not carried out by any of the authors. The physician in charge provided us with the radiographs and detailed information on all the applied surgical procedures. The first treatment involved the raising of a trapezoidal flap to remove the semi-impacted tooth 4.8, curettage of the cyst, and apicoectomies of 4.6 and 4.7. It should be noted that some mandatory surgical steps with these molars were not done, such as preparing and making retrograde filling of the cavities – these steps being essential for ensuring correct three-dimensional apical sealing. This could lead to the appearance of periapical periodontitis that may be confused with possible relapse of the cyst. With regard to the first treatment of the cyst, it is considered that reaching all the limits of such a large cavity could not be achieved, and that some cystic fragments probably remained in the surgical bed. For this reason, although integrity of the cyst was temporarily altered and a decompressive effect was achieved with centripetal bone regeneration thanks to removal of tooth 4.8, creating a communication with the oral cavity and performing curettage of the cyst, localized relapse occurred two years later in the posterior area of the body of the mandible. With respect to the design of the flap that was employed in the area of the oral mucosa that covered the presumed fenestration of teeth 4.6-4.7, it was trapezoidal in shape, and it can be assumed that with its elevation a part of the cyst or its epithelial remnants were fragmented and detached, leaving a small portion adhered inside the internal surface of the flap. This would explain why correct regeneration was not observed on the follow-up controls and that one year later a large recurrence was identified, manifesting with inflammation in the depth of the buccal sulcus. This new lesion was subjected to incision and drainage, with a clear decompression effect. However, persistence of its remains perpetuated the lesion, which was not treated until years later, simultaneously to curettage of the large recurrence in the ascending ramus of the mandible. Curiously, this relapse in the ascending ramus, which was larger in size, did not reappear again later on.

The above-mentioned data seem to evidence that OKC recurrence is due, among other reasons, to the persistence of cell remnants in the surgical bed and adjacent soft tissues in contact with the cyst, where these cells maintain their growth potential and thus constitute the cause of relapse.

Lastly, the slow growth of the latest relapse in the last few years may suggest that as a consequence of the repeated interventions on the same area, inflammation of the cyst wall occurred, inducing epithelial changes and a decrease in its mitotic activity. There may also have occurred changes in the stromal component as a consequence of the previous interventions, making it more fibrous, thus reducing vascularization and the arrival of growth factors needed for epithelial proliferation (10,26).

Thus, considering the three possible recurrence mechanisms described above, treatment from the start should focus on elimination of the possible vital epithelial cells that may remain after curettage, whether these are remnants of the original cyst or of satellite cysts left behind in the bone (9). Peripheral ostectomy or the cauterizing effect of fixating agents such as Carnoy’s solution or the effect of 5-FU in the bone bed should be enough to eliminate these cells. On the other hand, in cases with bone fenestrations allowing contact between the cyst wall and the mucosal soft tissues, exeresis of the overlying oral mucosa should be performed or, alternatively electro-cauterization can be done (12). This would eliminate any possible epithelial fragments or satellite cysts of the OKC unintentionally displaced as a result of raising of the flap during surgery, as well as any basal cell hamartomas of the oral mucosa that could give rise to new cysts. In relation to this, it should be mentioned that there have been reports of OKC recurrence in autologous bone grafts used for reconstruction following segmental resection of the lesion. This points out the importance of removing or electro-cauterizing the soft tissues in contact with the cyst (22). In addition, in the case of large OKCs with multiple bone perforations and in close contact with neighboring teeth or important anatomical structures, prior marsupialization/decompression is advised in order to reduce the size of the lesion and facilitate subsequent enucleation/curettage (10).

In our patient, the fourth and last recurrence was first subjected to marsupialization in order to reduce the size of the lesion and to increase its distance from the IDC. Having done this, removal of the entire oral mucosa overlying the buccal cortical fenestration was perfomed, as it could contain a portion of the cyst or potential sources of recurrence (satellite cysts or basal cell hamartomas). Following decompression, lesion curettage was performed and complemented with peripheral ostectomy, due to the multiple compartments found in the cavity and which complicated elimination. Lastly, in order to destroy any possible remaining fragments, chemical fixation with Carnoy’s solution was carried out. Although we believe that the implicated teeth could be preserved with this treatment approach, they were removed due to the poor periodontal support of 4.7 and the asymptomatic vertical root fracture of 4.6.

## Conclusions

• Odontogenic keratocysts are benign maxillary lesions with an aggressive biological behavior.

• The main purpose of surgical treatment is to cause the least damage possible while also reducing the possibility of recurrence as far as possible. Although radical treatment is probably able to reduce the risk of relapse, absolute certainty of cure cannot be achieved with a single surgical intervention, and the patients must undergo permanent radiological follow-up on a yearly basis.

• Marsupialization/decompression in a first phase offers many advantages. On one hand, it reduces the size of the cyst and induces bone regeneration; moves away the lesion from vital structures and reduces the risk of iatrogenic complications and facilitates subsequent enucleation/curettage when the capsule thickens. On the other hand, by performing the window at the site of bone perforation in marsupialization/decompression, the overlying oral mucosa is eliminated, thereby avoiding the possible development of new cysts arising from basal cell hamartomas or persistent epithelial fragments or satellite cysts of the OKC adhered to the soft tissues.

• Lastly, although enucleation/curettage after marsupialization/decompression should be sufficient, small epithelial fragments or satellite cysts may remain in the bone cavity. The use of peripheral ostectomy, cryotherapy, or the application of Carnoy’s solution or 5-FU, appears to reduce this risk.

In our opinion, there is no need for radical segmental bone resection in the management of OKCs, unless there is carcinomatous degeneration of the cyst or in the presence of relapse involving structures such as the pterygoid muscles and/or masseter muscle.

## Data Availability

The datasets used and/or analyzed during the current study are available from the corresponding author.

## References

[1] Borrás-Ferreres J, Sánchez-Torres A, Alberdi-Navarro J, Aguirre-Urizar JM, Mosqueda-Taylor A, Gay-Escoda C (2020). Therapeutic management of the odontogenic keratocyst. An energetic approach with a conservative perspective and review of the current therapeutic options. J Clin Exp Dent.

[2] Slusarenko da Silva Y, Stoelinga PJW, Naclério-Homem MDG (2019). The presentation of odontogenic keratocysts in the jaws with an emphasis on the tooth-bearing area: A systematic review and meta-analysis. Oral Maxillofac Surg.

[3] MacDonald-Jankowski DS (2011). Keratocystic odontogenic tumour: Systematic review. Dentomaxillofac Radiol.

[4] Stoelinga PJ (2001). Long-term follow-up on keratocysts treated according to a defined protocol. Int J Oral Maxillofac Surg.

[5] MacDonald-Jankowski DS, Li TK (2010). Keratocystic odontogenic tumour in a Hong Kong community: The clinical and radiological features. Dentomaxillofac Radiol.

[6] Al-Moraissi EA, Pogrel MA, Ellis E 3rd (2016). Enucleation with or without adjuvant therapy versus marsupialization with or without secondary enucleation in the treatment of keratocystic odontogenic tumors: A systematic review and meta-analysis. J Craniomaxillofac Surg.

[7] Slusarenko da Silva Y, Stoelinga PJW, Naclério-Homem MDG (2019). Recurrence of nonsyndromic odontogenic keratocyst after marsupialization and delayed enucleation vs. enucleation alone: A systematic review and meta-analysis. Oral Maxillofac Surg.

[8] Voorsmit RA, Stoelinga PJ, van Haelst UJ (1981). The management of keratocysts. J Maxillofac Surg.

[9] Stoelinga PJ (2005). The treatment of odontogenic keratocysts by excision of the overlying, attached mucosa, enucleation, and treatment of the bony defect with Carnoy solution. J Oral Maxillofac Surg.

[10] Pogrel MA (2015). The keratocystic odontogenic tumour (KCOT)--an odyssey. Int J Oral Maxillofac Surg.

[11] Díaz-Belenguer Á, Sánchez-Torres A, Gay-Escoda C (2016). Role of Carnoy's solution in the treatment of keratocystic odontogenic tumor: A systematic review. Med Oral Patol Oral Cir Bucal.

[12] Stoelinga PJW (2022). The odontogenic keratocyst revisited. Int J Oral Maxillofac Surg.

[13] Pogrel MA (2003). Decompression and marsupialization as a treatment for the odontogenic keratocyst. Oral Maxillofac Surg Clin North Am.

[14] Fidele NB, Bing L, Sun Y, Wu T, Zheng Y, Zhao Y (2019). Management of mandibular odontogenic keratocyst through radical resection: Report of 35 cases. Oncol Lett.

[15] Schmidt BL, Pogrel MA (2001). The use of enucleation and liquid nitrogen cryotherapy in the management of odontogenic keratocysts. J Oral Maxillofac Surg.

[16] Karaca C, Dere KA, Er N, Aktas A, Tosun E, Koseoglu O T (2018). Recurrence rate of odontogenic keratocyst treated by enucleation and peripheral ostectomy: Retrospective case series with up to 12 years of follow-up. Med Oral Patol Oral Cir Bucal.

[17] Caminiti MF, El-Rabbany M, Jeon J, Bradley G (2021). 5-Fluorouracil is associated with a decreased recurrence risk in odontogenic keratocyst management: A retrospective cohort study. J Oral Maxillofac Surg.

[18] Mosqueda-Taylor A (2008). New findings and controversies in odontogenic tumors. Med Oral Patol Oral Cir Bucal.

[19] Brito-Mendoza L, Bologna-Molina R, Irigoyen-Camacho ME, Martinez G, Sánchez-Romero C, Mosqueda-Taylor A (2018). A comparison of Ki67, Syndecan-1 (CD138), and molecular RANK, RANKL, and OPG triad expression in odontogenic keratocyts, unicystic ameloblastoma, and dentigerous cysts. Dis Markers.

[20] Harris M, Toller P (1975). The pathogenesis of dental cysts. Br Med Bull.

[21] Woolgar JA, Rippin JW, Browne RM (1987). A comparative histological study of odontogenic keratocysts in basal cell naevus syndrome and control patients. J Oral Pathol.

[22] Stoelinga PJW, Slusarenko da Silva Y (2021). The significance of recurrent odontogenic keratocysts in bone grafts. Int J Oral Maxillofac Surg.

[23] Tabrizi R, Hosseini Kordkheili MR, Jafarian M, Aghdashi F (2019). Decompression or marsupialization; which conservative treatment is associated with low recurrence rate in keratocystic odontogenic tumors? A systematic review. J Dent (Shiraz).

[24] Stoelinga PJ (2003). Etiology and pathogenesis of keratocysts. Oral Maxillofac Surg Clin North Am.

[25] Zhao YF, Wei JX, Wang SP (2002). Treatment of odontogenic keratocysts: A follow-up of 255 chinese patients. Oral Surg Oral Med Oral Pathol Oral Radiol Endod.

[26] Consolo U, Setti G, Tognacci S, Cavatorta C, Cassi D, Bellini P (2020). Histological changes in odontogenic parakeratinized keratocysts treated with marsupialization followed by enucleation. Med Oral Patol Oral Cir Bucal.

